# Progress in antileishmanial drugs: Mechanisms, challenges, and prospects

**DOI:** 10.1371/journal.pntd.0012735

**Published:** 2025-01-03

**Authors:** Haoran Zhang, Ruixi Yan, Yahui Liu, Mengtao Yu, Ziyi He, Junfeng Xiao, Kaijie Li, Gang Liu, Qin Ning, Yan Li

**Affiliations:** 1 Department of Infectious Disease, Tongji Hospital, Tongji Medical College and State Key Laboratory for Diagnosis and Treatment of Severe Zoonotic Infectious Diseases, Huazhong University of Science and Technology, Wuhan, China; 2 Department of Pathogen Biology, School of Basic Medicine, Tongji Medical College and State Key Laboratory for Diagnosis and Treatment of Severe Zoonotic Infectious Diseases, Huazhong University of Science and Technology, Wuhan, China; 3 Hubei Provincial Center for Disease Control and Prevention, Wuhan, China; 4 College of Veterinary Medicine, China Agricultural University, Beijing, China; 5 Department and Institute of Infectious Disease, Tongji Hospital, Tongji Medical College and State Key Laboratory for Diagnosis and Treatment of Severe Zoonotic Infectious Diseases, Huazhong University of Science and Technology, Wuhan, China; 6 Department of Pediatrics, Tongji Hospital, Tongji Medical College, Huazhong University of Science and Technology, Wuhan, Hubei, China; Uniformed Services University: Uniformed Services University of the Health Sciences, UNITED STATES OF AMERICA

## Abstract

Leishmaniasis, a neglected tropical disease caused by *Leishmania* parasites, continues to pose global health challenges. Current treatments face issues like resistance, safety, efficacy, and cost. This review covers the discovery, mechanisms of action, clinical applications, and limitations of key antileishmanial agents: pentavalent antimonials, amphotericin B, miltefosine, paromomycin, and pentamidine. Despite toxicity and resistance (antimonials), hospitalization needs and side effects (amphotericin B), regional efficacy variability (miltefosine), inconsistent outcomes (paromomycin), and severe side effects (pentamidine), these drugs are vital. Novel strategies to overcome the deficiencies of current therapies are highlighted, including combination regimens, advanced drug delivery systems, and immunomodulatory approaches. Comprehensive and cooperative efforts are crucial to fully realize the potential of advancements in antileishmanial pharmacotherapy and to reduce the unacceptable worldwide burden imposed by this neglected disease.

## Introduction

Leishmaniasis, caused by the *Leishmania* species within the *Trypanosomatidae* family, is a significant global health challenge, with about 20 pathogenic species transmitted via sandfly bites [1,2]. The disease manifests in various forms, including visceral, mucosal, cutaneous, and mucocutaneous leishmaniasis (VL, ML, CL, and MCL), with severity influenced by the *Leishmania* strain and host immune response [3,4]. Outcomes range from self-healing cutaneous lesions to potentially fatal visceral diseases, illustrating the intricate parasite–host interactions [1]. Macrophages, pivotal in disease pathogenesis, are the primary host cells for *Leishmania*, highlighting complex parasite–host dynamics [5]. Despite significant research efforts, leishmaniasis continues to present challenges, including drug resistance and restricted access to treatments, which underscores the urgent need for novel therapeutic approaches.

Addressing these challenges requires improved drug therapy, as current treatments face issues such as toxicity, resistance, and limited availability, particularly in resource-poor regions. Initially, pentavalent antimonials (sodium stibogluconate and meglumine antimoniate) were primary treatments [1], but resistance, especially in regions like India, has reduced their use [6]. Amphotericin B, though effective, carries significant toxicities [7–9]. Liposomal amphotericin B, FDA-approved for leishmaniasis, is hampered by logistical issues in low-resource settings [10]. Miltefosine has been approved for the treatment of leishmaniasis [11], but other drugs like paromomycin and pentamidine are still in use. However, these treatments face significant challenges, such as resistance, high costs, and severe side effects [12], highlighting the pressing need for safer and more effective therapeutic options.

Our review discusses various currently available therapeutic strategies regarding leishmaniasis treatment, delving into the molecular mechanisms and evaluating the merits and drawbacks of mainstream drugs, including pentavalent antimonials, amphotericin B, miltefosine, paromomycin, and pentamidine. Being one of the most dangerous Neglected Tropical Diseases NTDs, leishmaniasis requires immediate attention and cutting-edge treatment approaches to overcome its multifaceted challenges. This requirement sets the setting for the following sections, where we will discuss new treatments and how to improve the ones that already exist.

## Methods

A comprehensive literature search was conducted using databases such as PubMed, Scopus, Google Scholar, and Web of Science to identify relevant articles published from 2000 to March 2024 on antileishmanial drug discovery and development. Keywords used in the search included “leishmaniasis,” “antileishmanial drugs,” “drug resistance,” “treatment,” and “novel therapies.” The search was limited to articles published in English. Titles and abstracts were screened for relevance, and full-text articles were reviewed to extract key information on the mechanisms of action, clinical efficacy, and limitations of current treatments. Additional sources were identified through the reference lists of selected articles.

### 1. Pentavalent antimonial

Pentavalent antimonial compounds, including sodium stibogluconate and meglumine antimoniate, have been the cornerstone of leishmaniasis treatment for over 70 years. Discovered in the early 20th century, antimony potassium tartrate marked a significant advancement in antileishmanial therapy [13]. Despite its initial success against VL in various regions, the treatment faced setbacks due to high toxicity, lengthy treatment durations, and emerging parasite resistance [14]. A turning point came in 1947 with the introduction of the less toxic sodium stibogluconate, achieving up to 90% cure rates [15]. Even in recent years, pentavalent antimonials remain vital, albeit with growing resistance concerns [16,17].

#### 1.1 Molecular mechanism of the inhibition of *Leishmania* by pentavalent antimony

**Fig 1:**
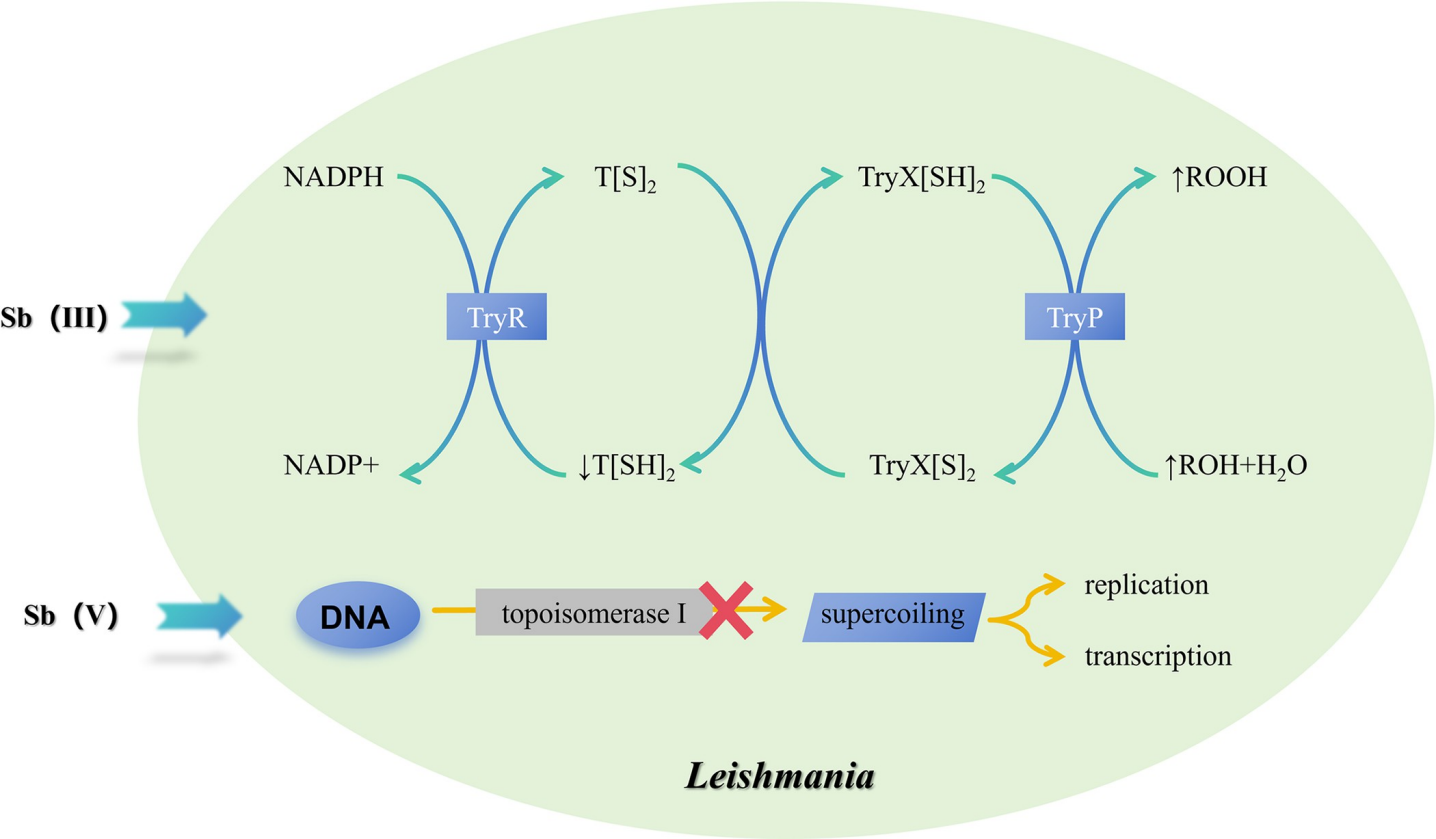
Mechanism of action of pentavalent antimonials in Leishmania. Upon administration, pentavalent antimony (Sb(V)) is bioreduced to trivalent antimony (Sb(III)) within *Leishmania* parasites, leading to 2 primary pathways of action. Sb(III) inhibits trypanothione reductase (TryR), disrupting the parasite’s redox balance and increasing oxidative stress. Concurrently, Sb(III) affects DNA topoisomerase I activity, impairing DNA supercoiling essential for replication and transcription. Additionally, Sb(V) shows intrinsic antileishmanial activity independent of its reduction to Sb(III), further complicating the parasite’s survival. Trypanothione peroxidase (TryP) participates in detoxifying reactive oxygen species (ROS), and the inhibition of TryR enhances ROS generation, leading to further damage to parasite macromolecules, including DNA and proteins, disrupting homeostasis and contributing to parasite death.

The antileishmanial activity of pentavalent antimonial (Sb(V)) compounds is understood through multiple interconnected mechanisms (Fig 1). Initially considered a prodrug, Sb(V) is bioreduced within *Leishmania* parasites to yield the trivalent form (Sb(III)), which directly imparts antiparasitic effects [18]. Sb(III) forms stable complexes with thiol groups, particularly glutathione in mammalian cells [19,20] and trypanothione in trypanosomatid parasites [21–23], disrupting critical cellular processes. Exposure to pentavalent antimonials prompts *Leishmania* to activate multidrug resistance transporters, expelling Sb(III)-thiol complexes and leading to thiol depletion, oxidative stress, and apoptosis [24–27]. Moreover, Sb(III) inhibits the trypanothione reductase system, pivotal for maintaining the intracellular redox balance within trypanosomatids, further exacerbating oxidative damage [28–30]. Crystallographic evidence elucidates the interaction of Sb(III) with the trypanothione reductase enzyme, underscoring a direct mechanism of action [31]. Moreover, pentavalent antimonials exhibit intrinsic antileishmanial effects, independent of bioreduction to Sb(III). Sodium stibogluconate and ureastibamine disrupt DNA topoisomerase I activity in *Leishmania donovani*, impeding DNA supercoiling, essential for replication and transcription [32,33]. Investigations also show Sb(V) forming stable complexes with adenine nucleosides, suggesting interference with nucleic acid metabolism [34]. These findings highlight the dual action of Sb(V), combining the generation of bioactive Sb(III) within cells and direct disruption of vital parasitic functions. Moreover, research on leishmaniasis and pentavalent antimonial compounds (such as SSG) also demonstrated that SSG affects the immune system [35]. This comprehensive approach, including both prodrug conversion and direct antileishmanial activity, illustrates the complexity of Sb(V)’s mechanism against *Leishmania*, offering insights for overcoming drug resistance and enhancing treatment efficacy.

#### 1.2 Current clinical applications of pentavalent antimonials

Pentavalent antimonial drugs, encompassing meglumine antimoniate (also known as Glucantime) and sodium stibogluconate (also called Pentostam), have historically been pivotal in treating all primary leishmaniasis forms, including CL, MCL, and VL [14,36,37]. Their use as the premier choice for CL and ML treatments stems from extensive clinical validation and their influence on the evolution of novel formulations and combination therapies [16]. According to the WHO 2010 guidelines, pentavalent antimonials are among the first-line treatment options for certain species of *Leishmania*, such as *L*. *aethiopica* in Old World CL. In the Indian subcontinent and East Africa, despite being administered intramuscularly or intravenously at a standard dosage of 20 mg Sb(V) per kg body weight daily over roughly a month, resistance in some regions has necessitated the search for optimized protocols and alternatives, underscoring the drug’s variable success and the pressing need for new strategies to address toxicity, resistance, and accessibility [1,6]. However, for other species like *L*. *major*, alternative therapies such as fluconazole are recommended as first-line treatments, particularly in North Africa, as endorsed by ASTMH/IDSA guidelines [38,39].

While pentavalent antimonial compounds initially showed promising therapeutic efficacy against leishmaniasis, varied clinical outcomes over time across different regions have become evident [40]. The side effects associated with pentavalent antimony are generally mild, including injection site pain, arthralgia, reversible peripheral neuropathy, and gastrointestinal discomfort. However, patients with HIV co-infection have a heightened risk of developing pancreatitis [41,42]. Prolonged use of higher doses has been linked to severe toxicities such as liver and renal failure, with some instances of significant cardiotoxicity, characterized by inverted T-waves, extended QTc interval on ECG, and potentially fatal arrhythmias [42–45]. The emergence of resistance to antimonial treatments significantly challenges their efficacy, with resistance leading to suboptimal outcomes and persistent infections, notably in India where failure rates surged dramatically between 1980 and 1997, reaching up to 65% in some areas [6,37,46]. The issue of drug resistance has prompted extensive research by scientists. Walker and colleagues found that S-adenosylmethionine synthetase (SAMS) and S-adenosylhomocysteine hydrolase (SAHH) were overexpressed in Sb(III)-resistant lines and isolates, which is the key molecule in Sb-resistance in *Leishmania* [47]. Analysis showed that *Leishmania* parasites overexpressing LABCG2 were resistant to antimony due to reduced Sb(III) accumulation via increased efflux. LABCG2 also transported thiols in the presence of Sb(III), as confirmed by biotinylation assays [48]. This underscores the need for new therapeutic options and optimized treatment protocols to counteract resistance and uphold the utility of pentavalent antimonials against leishmaniasis.

Several approaches have been explored to address the limitations of conventional pentavalent antimony therapy, including optimizing treatment protocols and combining therapies. For example, local antimony injections into CL lesions, endorsed by WHO, reduce side effects [49,50], and combination with cryotherapy shows efficacy against CL [16,51]. For VL, pentavalent antimony combined with other drugs like paromomycin has improved outcomes in Africa [52]. Sb(V) oxide and its complexes spontaneously form nanoaggregates or micelles in water, making it feasible to design new Sb(V) complexes with supramolecular assemblies for treating leishmaniasis effectively [53].

Beyond protocol optimization, advancing novel pentavalent antimony formulations is pivotal for enhancing antileishmanial efficacy. Liposome encapsulation improves solubility and targeted delivery [54,55], and designing amphiphilic Sb(V) molecules aims at better oral absorption for VL treatment [56]. Additionally, cyclodextrin complexes are developed to increase oral bioavailability [57]. These innovations aim to maintain pentavalent antimony’s clinical relevance through new combination regimens, administration methods, and addressing toxicity, resistance, and delivery challenges.

### 2. Amphotericin B

Amphotericin B (AmB), discovered in 1955 from Streptomyces nodosus, has been a cornerstone in treating serious fungal infections and shows broad activity against various pathogens including yeasts, dimorphic fungi, and molds [58,59]. Its antileishmanial potential was recognized early, with in vitro effectiveness established in 1960 and the first successful clinical use against VL reported in 1963 [8,9]. The primary challenge with AmB’s use is its insolubility in water, leading to the adoption of a nephrotoxic deoxycholate form as a second-line treatment for VL, CL, and MCL since the 1960s [60–62]. The development of liposomal delivery systems in the 1970s facilitated the creation of AmBisome, a liposomal formulation with improved bioavailability and reduced toxicity [62,63]. Although resistance to AmB in *Leishmania* species was historically considered a minor concern, emerging strains indicate potential patient hazards [64,65].

#### 2.1 Molecular mechanism of the inhibition of *Leishmania* by amphotericin B

**Fig 2:**
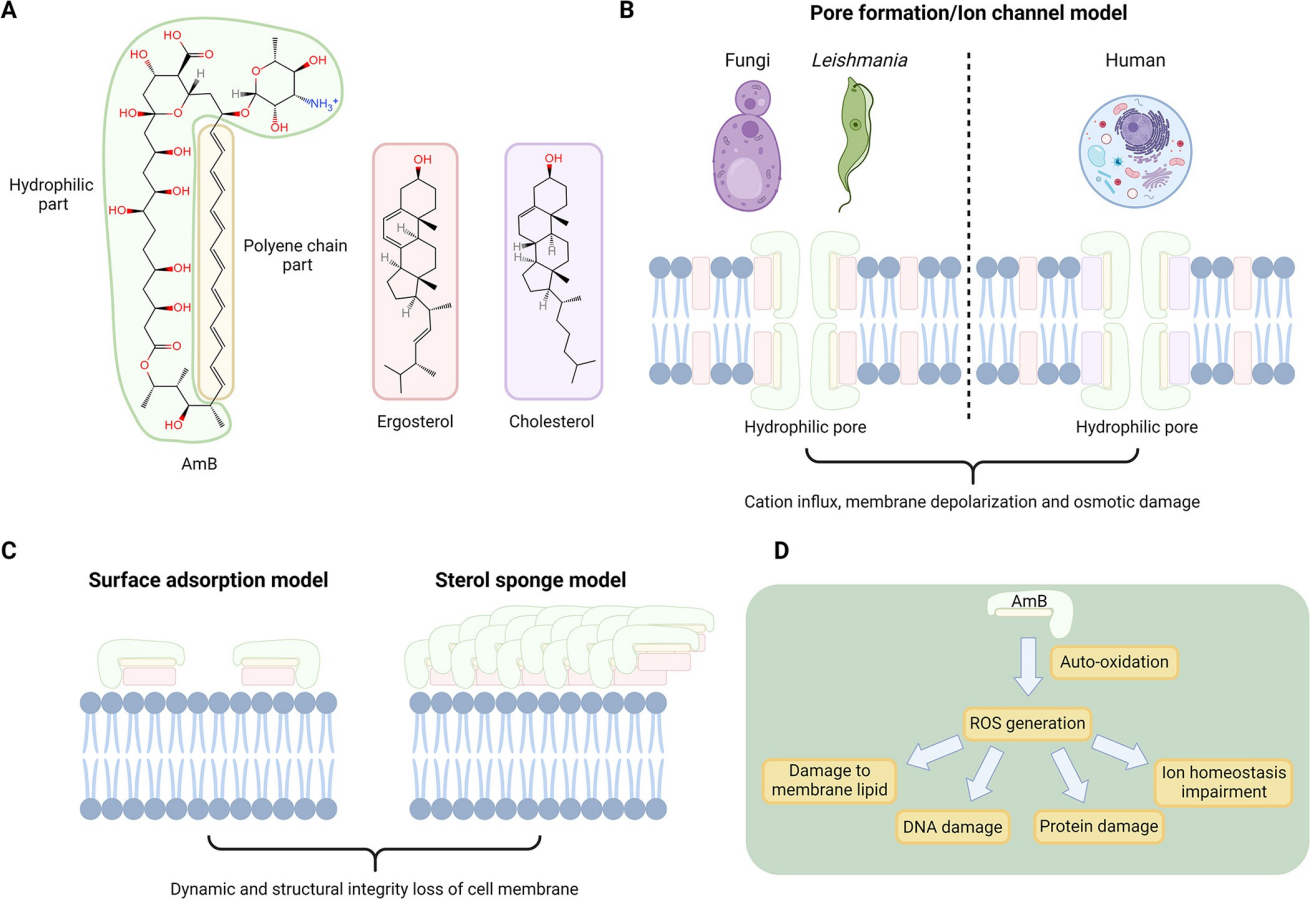
Chemical structure and proposed action mechanism of amphotericin B (AmB) against *Leishmania* parasites. (A) The chemical structure of AmB, highlighting its polyene core that binds the membrane sterol ergosterol in fungi and *Leishmania*, as well as cholesterol in human. (B) The classical pore formation/ion channel model proposes AmB incorporated into the sterol-rich membrane, forming aqueous cytotoxic pores. (C) The ergosterol extraction mechanism is characterized by an alternative surface adsorption model and a sterol sponge model. (D) AmB-induced ROS generation, a result of auto-oxidation, further damages the parasite by targeting membrane lipids, DNA, proteins, and disrupting ion homeostasis, contributing to parasite cell death. Created with Biorender.com.

AmB exerts its potent antileishmanial effects predominantly through binding ergosterol in *Leishmania* and fungi cell membranes [66,67] significantly stronger than cholesterol in human cells [68,69], highlighting its preferential affinity that’s critical for its action. This preferential interaction is facilitated through hydrogen bonding and van der Waals forces [64,70], where AmB’s configurational compatibility with ergosterol, notably at C7, C22 double bonds, and C24 side-chain methylation [60,64], enhances its selective toxicity towards *Leishmania* by forming ion channels or lipid aggregates on membranes [61,70], leading to cell death through osmotic imbalance and ion homeostasis disruption. Recent studies further elucidate AmB’s mechanism, suggesting that beyond ion channel formation, AmB may aggregate on membrane surfaces to extract essential lipids, directly leading to *Leishmania* cell death [71], aligning with the sterol sponge model [72–74]. Additionally, structural studies using NMR and molecular dynamics simulations have revealed that AmB assembles into stable seven-molecule ion channels when interacting with ergosterol [75]. This formation, while established in fungal membranes, may also suggest a potential role in disrupting ergosterol-rich *Leishmania* membranes. Collectively, AmB has strong antileishmanial effects via intricate processes (Fig 2).

#### 2.2 Current clinical applications of amphotericin B

Amphotericin B’s clinical application is substantially constrained by its poor water solubility and low oral bioavailability, driving the exploration of alternative formulations to circumvent these limitations [76,77]. Due to its molecular size, AmB tends to precipitate in acidic environments, resulting in an oral bioavailability of merely 0.3% [78,79]. Consequently, the deoxycholate form of AmB, which is more soluble, necessitates inconvenient hospitalization for intravenous administration due to its inherent toxicity [80,68]. To address these challenges, liposomal formulations, particularly AmBisome, have been developed. By encapsulating AmB within phospholipid bilayers, these formulations improve drug distribution to tissues and enhance plasma levels, which significantly reduces toxicity while retaining AmB’s efficacy against *Leishmania* [63,81–84]. Recent research emphasizes the importance of optimizing the physicochemical properties of liposomal AmB formulations for improved treatment outcomes in both cutaneous and visceral leishmaniasis, with ongoing advancements in topical and oral liposomal AmB formulations being explored [85]. Additionally, the development of pH-sensitive nanostructured lipid carriers (AmB-NLCs) has demonstrated promising results, with enhanced drug release under acidic conditions, potentially offering a targeted approach for localized leishmaniasis treatment [86]. This approach not only mitigates the side effects associated with traditional AmB but also enhances its therapeutic effectiveness, particularly in treating visceral leishmaniasis, underscoring the importance of advancements in drug delivery systems for leishmaniasis treatment [87,88]. Liposomal formulations have thus emerged as critical alternatives to traditional AmB, providing effective treatment options in regions with high leishmaniasis prevalence.

AmB became the primary therapy in Bihar, India, in the 1990s, addressing resistance to first-line drugs [70]. Its effectiveness is notable, but use is restricted due to the need for prolonged hospitalization and nephrotoxicity [62,68,80,89]. Treatment protocols vary, with dosages ranging from 7 to 20 mg/kg, potentially requiring up to 43 days to achieve near 100% cure rates against both antimony-sensitive and refractory cases [90]. Liposomal AmB (L-AmB) was introduced to reduce these drawbacks, leading to shorter hospital stays and improved outcomes [91–94]. A study in India reported a 95.7% cure rate with a single 10 mg/kg dose of L-AmB [95], prompting the WHO to recommend it as the first-line treatment in South Asia [96].

L-AmB enhances drug delivery to organs, allowing for high doses with less kidney damage [92] and nearly 100% cure rates [93]. This formulation has proven effective in both children and adults across various regions, including the Mediterranean, the Middle East, and Brazil, with doses of 20 mg/kg [97,98]. The Pan American Health Organization endorses L-AmB as the primary VL treatment, with 3 to 5 mg/kg doses showing up to 100% success in southern Europe [99]. While effective against VL, outcomes for ML and disseminated disease vary [87]. Topical L-AmB gel emerges as a new option for localized CL, offering an alternative to low-efficacy topicals and systemic treatments with toxicity risks [100]. A recent study showed mild local adverse reactions in less than 30% of CL patients [100].

Clinical applications have revealed variable AmB efficacy against different *Leishmania* strains and clinical manifestations. Uruguayan outbreak isolates associated with VL demonstrated higher infectivity and reduced drug sensitivity compared to South American reference strains [101]. In French Guiana, CL among military personnel showed significant treatment failures with pentamidine and L-AmB, necessitating alternative treatments [102]. A case of imported CL caused by *Leishmania infantum* in Korea was successfully treated with liposomal AmB [94]. The main concerns with AmB include its nephrotoxicity and severe infusion-related side effects, such as renal insufficiency and metabolic disorders [62,103], contrasting with L-AmB’s fewer adverse effects [94]. Advances have led to AmB derivatives with reduced renal toxicity and preserved antifungal efficacy [104], though their potential against diverse *Leishmania* strains remains under evaluation. Drug resistance is another challenge [105]; for example, in vitro studies indicate that *L*. *infantum* strains associated with VL in dogs showed resistance following miltefosine-allopurinol treatments, which also conferred cross-resistance to AmB [106]. Additionally, reports from Brazil have highlighted resistance to AmB in *L*. *amazonensis* strains, which are associated with CL, underscoring the need for vigilant resistance monitoring across different regions [107]. Similar AmB-miltefosine cross-resistance was observed in mutant and clinical relapse *L*. *martiniquensis* strains, affecting both VL and CL cases [108].

A significant drawback of the novel, commercial, low-toxicity amphotericin B lipid formulation is the economic burden. This product has a much higher price than the conventional amphotericin B deoxycholate, which limits its accessibility and affordability for many patients [109]. As the high expense of L-AmB hinders widespread use, costs are lowered in India with the 10 mg/kg single L-AmB dose scheme [95]. Further optimization continues on delivery matrices like chitosan and dendrimer nanoparticles to improve amphotericin B solubility, release, and toxicity against *Leishmania major* [110]. Combination strategies with short-course miltefosine may also enhance efficacy compared to miltefosine alone against VL spread in India [111].

### 3. Miltefosine

Miltefosine, an alkylphosphorylcholine compound with broad-spectrum antitumor, antiparasitic, and antifungal properties [112], is the only oral agent currently approved for leishmaniasis treatment, representing a significant advantage over injectable alternatives [113]. Initially developed as an anticancer drug, its efficacy against *Leishmania* parasites was serendipitously discovered in the late 1980s, marking it as a promising candidate for both VL and CL treatment [114]. Its unique oral administration convenience underscores miltefosine’s pivotal role in advancing leishmaniasis treatment modalities [11].

#### 3.1 Molecular mechanism of the inhibition of *Leishmania* by miltefosine

Miltefosine is a pleiotropic drug with multiple targets [115]. The research indicates that miltefosine may involve interfere with parasite lipid metabolism, induce programmed apoptosis-like death, modulate host immunity, and disrupt mitochondrial function [116]. Recent studies suggest that miltefosine may also exert antiparasitic effects by affecting calcium homeostasis in *Leishmania* [115,117]. However, miltefosine exerts its antileishmanial effects primarily through disrupting lipid metabolism within *Leishmania* parasites and interfering with their activation of host cell lipid signaling pathways, crucial for their survival. In the past, multiple hypotheses have been proposed for the mechanism of action about miltefosine in anti-*Leishmania* lipid metabolism [118]. For example, interfering with glycosylphosphatidylinositol (GPI) anchor biosynthesis and inhibiting glycosomal alkyl-specific acyl-CoA acyltransferase as the target of action to interfere with ether-phospholipid metabolism [119], but all have received challenges in recent years [120].

**Fig 3:**
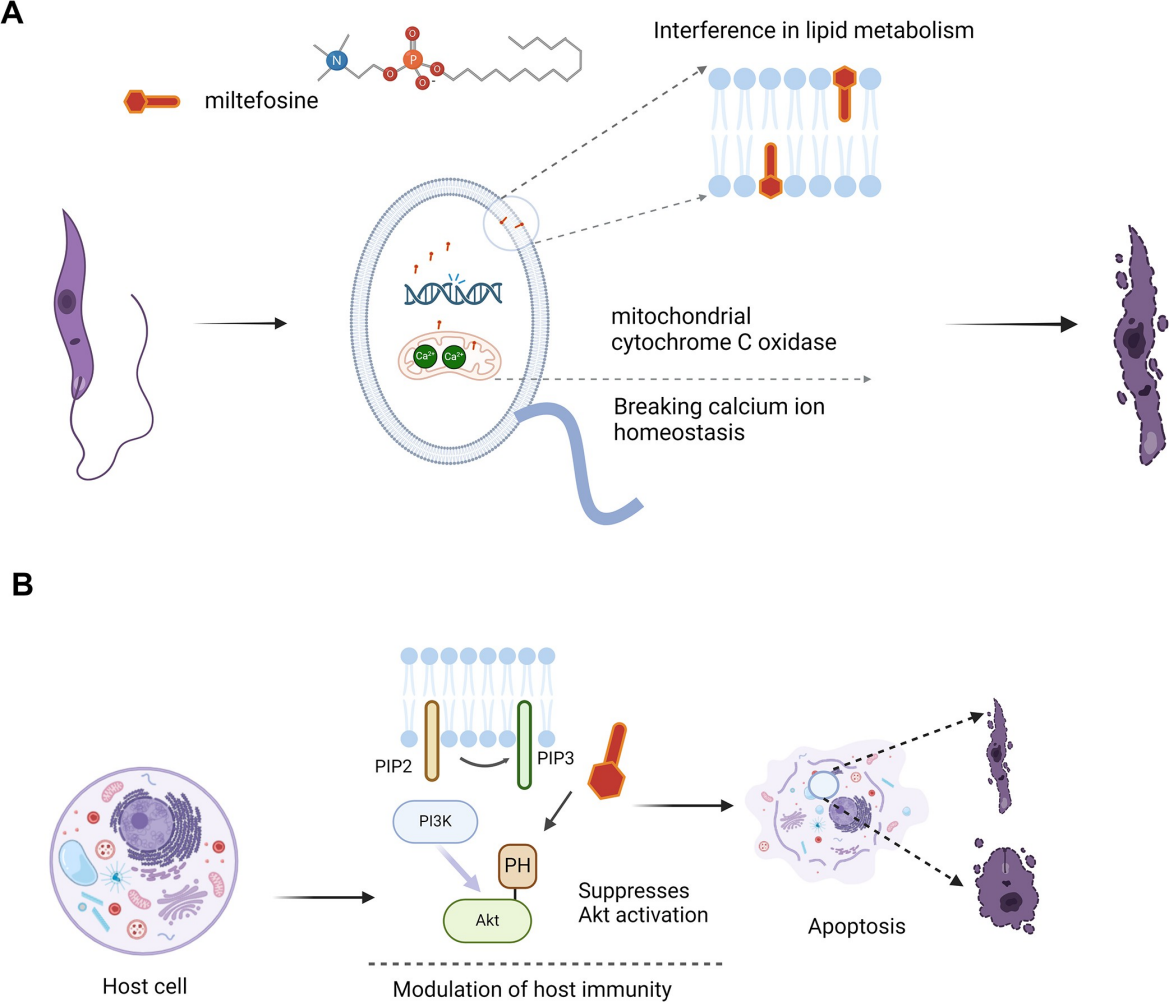
Mechanisms of anti-leishmanial action by miltefosine. (A) Miltefosine disrupts *Leishmania* parasites through multiple mechanisms. It interferes with lipid metabolism by integrating into parasite membranes, disrupts calcium ion homeostasis, and inhibits mitochondrial cytochrome C oxidase, collectively leading to parasite death. (B) In host cells, miltefosine modulates the immune response by affecting the PI3K/Akt signaling pathway, inducing apoptosis in infected cells, which further enhances its antiparasitic efficacy. Created with Biorender.com.

Specifically, it hampers the phosphatidylinositol 3-kinase (PI3K) signaling exploited by *Leishmania* to enter host cells and form an anti-apoptotic niche [121,122]. Miltefosine suppresses Akt activation, reversing PI3K-mediated survival signals, leading to apoptosis and infection control [123]. Though its exact target is unidentified, miltefosine likely competes with Akt pleckstrin homology (PH) domain for binding to PI(3,4)P2/PI(3,4,5)P3 phospholipids [124–126]. As a synthetic phosphatidylcholine analog, it is suggested to directly affect the parasite’s glycolipid, phospholipid, and sterol metabolism [118,127]. Omics profiling of miltefosine-treated *L*. *donovani* highlights its impact on biosynthesis pathways, underscoring the need for further research to detail its mechanisms and enhance treatment efficacy [128,129]. Thus, the multifaceted mechanism of action of miltefosine not only disrupts the basic survival pathway of *Leishmania* protozoa, but also has a modulatory effect on the immune system of the host (Fig 3). This emphasizes the imperative for in-depth study of miltefosine to further explore its therapeutic potential.

#### 3.2 Current clinical applications of miltefosine

Miltefosine, the only orally administered VL treatment to date [130], offers clear advantages over injectables. It is seen as safe, with mild gastrointestinal upset as the most common side effect [131]. Yet, its use in pregnant patients presents teratogenic risks [132], and there have been instances of ocular complications in post-kala-azar dermal leishmaniasis (PKDL) [133,134]. Additionally, reversible male reproductive toxicity has been reported [135], indicating the necessity for additional investigations. Miltefosine’s global utilization spans all major leishmaniasis forms, including VL, CL, MCL, and PKDL [136–140], notably approved in India for oral VL therapy as Impavido in 2002 [141]. Endorsed by WHO for PKDL treatment in East Africa, Bangladesh, India, and Nepal [142,143], it is recommended at 2.5 mg/day for 28 days for CL [140]. Its efficacy against New World CL rivals sodium stibogluconate [1], though bioavailability and efficacy vary by region and leishmaniasis type, partly due to pharmacogenomic differences [144]. Moreover, the FDA’s approval is based on studies that have shown that the susceptibility of *Leishmania* to miltefosine varies by *Leishmania* species, strains of a *Leishmania* species, and different geographic regions [145,146]. Despite these challenges, miltefosine’s oral administration, affordability, and safety profile maintain its significant role in global leishmaniasis treatment.

Despite emerging resistance issues [1], miltefosine remains a key player in combination therapies for leishmaniasis, favored for its cost-effectiveness and patient compliance. It is notably less expensive than L-AmB [147], offering economic advantages for VL treatment in the Indian subcontinent [140]. However, it is worth noting that due to the embryotoxicity of miltefosine, a contraceptive coverage period of 2 to 5 months is required after miltefosine use in the potential population, depending on the duration of treatment, which will limit its overall effectiveness [144]. Combining miltefosine with paromomycin or amphotericin B enhances its efficacy [144], which significantly reduce treatment duration and costs, thereby improving adherence [1,148]. In detail, the combination regimen of L-AmB 5 mg/kg single dose plus miltefosine 2.5 mg/kg per day in the treatment of visceral leishmaniasis was able to shorten the classical 28-day treatment by miltefosine to 7 days [148]. Moreover, synergies with antimony sodium gluconate have been noted [149]. Recent advancements, like thermotherapy and metal nanoparticle-encapsulated miltefosine, are tackling the challenge of prolonged treatment durations, showing promise in early studies for boosting efficacy and reducing toxicity [144,150]. These innovations underline miltefosine’s enduring significance in antileishmanial pharmacotherapy, supported by new therapies and combination treatments that extend its applicability against leishmaniasis.

### 4. Paromomycin

Paromomycin, an antibiotic first isolated from Streptomyces krestomuceticus in the 1950s, is unique for its clinically significant antileishmanial properties [151]. Recognized for its antileishmanial potential since 1961 through murine studies [152], its effectiveness against VL was confirmed in human trials by the Kenya Medical Research Institute (KEMRI) in Nairobi and the Hospital for Tropical Diseases in London later in the 1990s [153,154]. This led the Institute for OneWorld Health to develop an intramuscular paromomycin sulfate formulation, approved in India in 2006 for affordable VL treatment [1,151,155,156].

#### 4.1 Molecular mechanism of the inhibition of *Leishmania* by paromomycin

**Fig 4:**
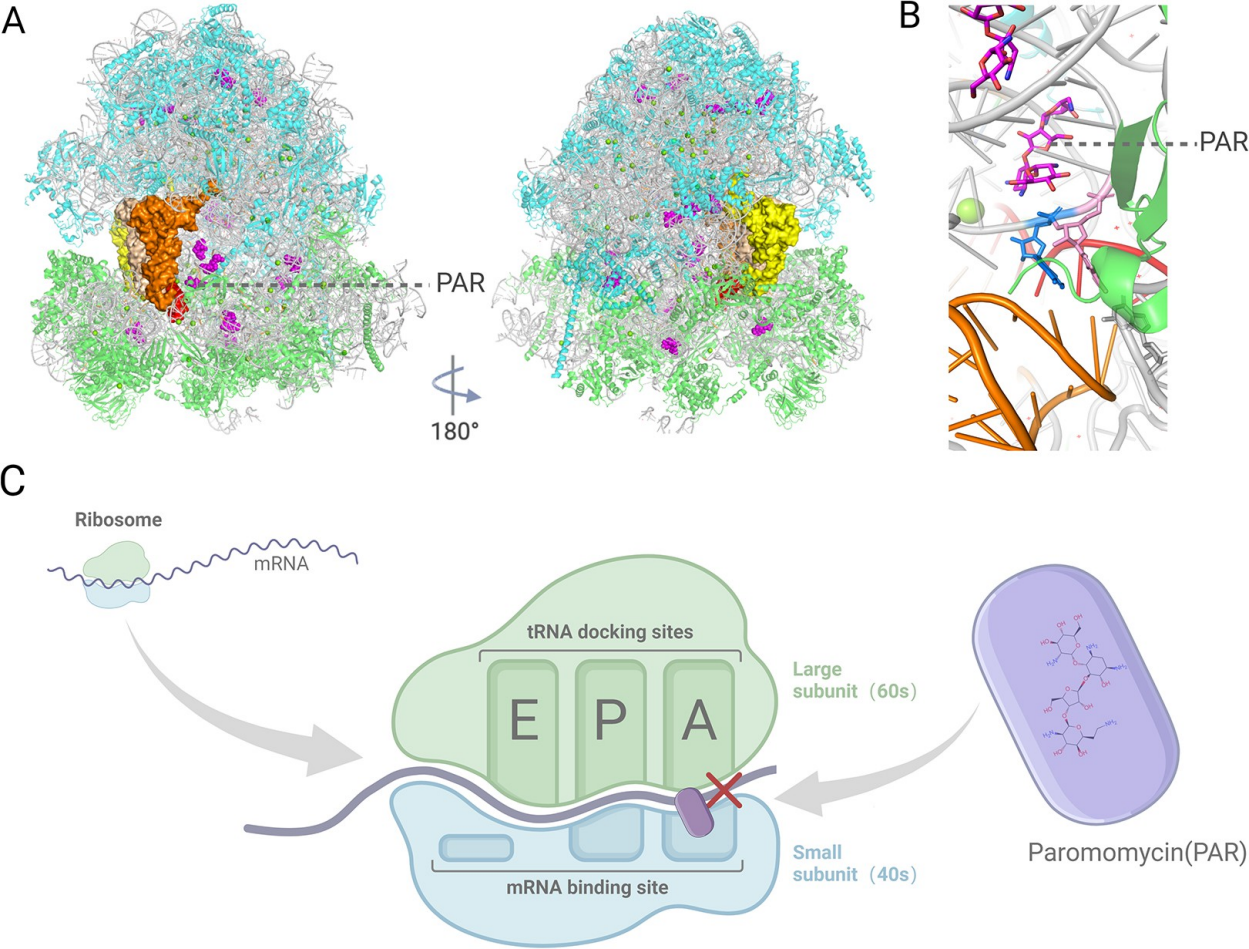
Paromomycin targets the decoding center of the *Leishmania* cytosolic ribosome. (A) A cryo-EM structure of the Leishmania cytosolic ribosome, showing 3 tRNAs positioned at the A-site (orange), P-site (beige), and E-site (yellow), with the mRNA in red. Paromomycin (PAR) is shown in purple, bound to the ribosomal RNA (rRNA), with ribosomal proteins in green and light blue representing the small (40S) and large (60S) subunits, respectively. (B) A close-up view of the PAR-binding pocket, highlighting its interaction with the decoding center of the ribosome, analogous to its binding site in bacterial ribosomes. (C) Paromomycin binds to the aminoacyl-tRNA recognition site on the small ribosomal subunit (40S), interfering with the translation process by causing mistranslation of the peptide chain, which compromises *Leishmania* growth. Created with Biorender.com.

Paromomycin’s antileishmanial activity primarily involves disrupting ribosomal function and mitochondrial membrane potential, affecting protein synthesis and energy metabolism [157–159]. It likely targets *Leishmania* protein translation by binding to ribosomal RNA, similar to its antibacterial effects [160,161], specifically disrupting peptide chain translation by binding to the 30S ribosomal subunit and interacting with 16S rRNA [162]. Further, paromomycin impedes translation by enhancing ribosomal subunit association and preventing dissociation [161], with RNA sequencing and experiments identifying inhibitory interactions [160,163]. Cryoelectron microscopy has shown paromomycin binds the 91S subunit, disrupting tRNA recruitment [164], and also disrupts mitochondrial respiration and membrane potential [159], illustrating its broad antileishmanial mechanism (Fig 4).

#### 4.2 Current clinical applications of paromomycin

Paromomycin, effective against VL and CL, is widely available and affordable, especially in endemic regions [156,165]. Its administration is mainly intramuscular for VL or topical for CL due to limited oral bioavailability [157,165]. The WHO recommended a paromomycin and sodium stibogluconate combination for VL in East Africa in 2010 [96], with subsequent studies validating up to 95% cure rates [166]. A 2015 Phase III trial in Bangladesh also highlighted a 94% efficacy of paromomycin monotherapy at 11 mg/kg for VL over 21 days, showing 94% efficacy and mild side effects [167]. In Israel since the 1990s, a topical ointment with 15% paromomycin and 12% reactive oxygen species has been utilized for CL, showing effectiveness against *L*. *major*, *L*. *panamensis*, *L*. *mexicana*, and *L*. *braziliensis* across various regions [168–171].

Nevertheless, the efficacy of paromomycin exhibits variability among different strains and populations [165]. Additionally, it is associated with adverse reactions such as localized pain at the injection site and transient auditory impairment, with a small percentage of patients experiencing nephrotoxic effects [156]. In light of these challenges, ongoing efforts focus on optimizing protocols and exploring novel delivery systems to enhance its risk-benefit ratio including the investigation of modified formulations such as 15% paromomycin with 10% urea [172] or 0.5% gentamicin [173,174] to mitigate adverse effects. Innovative paromomycin formulations like microspheres, liposomes, and hydrogels for both VL and CL are enhancing its efficacy and safety [175,165]. Khan and colleagues’ microsphere approach notably reduces nephrotoxicity [176], while liposomal formulations improve absorption and exhibit immunological benefits [177]. Solid lipid nanoparticles offer sustained release and reduced toxicity, increasing antileishmanial efficacy [178,179]. Biodistribution assays showed iontophoretic transport delivered higher PAR amounts to deeper skin layers than conventional ointment [180]. These advances are optimizing paromomycin’s therapeutic profile, promising to enhance its role in leishmaniasis treatment.

### 5. Pentamidine

Pentamidine, a synthetic amidine derivative synthesized in the late 1930s [181], initially treated VL before the 1950s [182] and later addressed drug-resistant CL in the 1970s [183]. Commercialized as an isethionate ester in 1984 [184,185], it now serves as a second-line option for leishmaniasis due to efficacy limits and toxicity [186]. As drug resistance escalates, elucidating pentamidine’s mechanisms of action and developing safer derivatives may unlock new possibilities for this old medication.

#### 5.1 Molecular mechanism of the inhibition of *Leishmania* by pentamidine

Pentamidine’s effects on *Leishmania* parasites are notably complex and not yet fully understood. It interacts with various nucleic acids, disrupting nucleotide incorporation and oxidative phosphorylation, thereby affecting the biosynthesis of DNA, RNA, phospholipids, and proteins. It disrupts the MBNL1-CUG repeat complex in DM1, affecting alternative splicing of pre-mRNAs [187], and shows broad RNA-binding activity, including interactions with CUG RNA repeats and intron stem-loop RNA [188]. Furthermore, pentamidine’s nonspecific tRNA binding interferes with aminoacylation processes [189], adding another layer to its multifaceted inhibitory effects. Pentamidine may bind to the kinetoplast DNA, inhibiting mitochondrial respiratory chain complex II, inducing apoptosis through increased intracellular calcium [185]. Its competing with polyamines [190] significantly inhibits polyamine synthesis, critical for purine-lacking *Leishmania* [191]. These interactions highlight the need for thorough evaluation of pentamidine’s target engagement and its promiscuous binding behavior [192].

#### 5.2 Current clinical applications of pentamidine

Previously a first-line leishmaniasis treatment, pentamidine’s use has declined due to adverse effects and new therapies, now serving mainly as a second-line option [186]. Its efficacy varies across *Leishmania* species, remaining a first-line recommendation for *L*. *guyanensis*-induced CL and MCL in several South American countries, backed by high cure rates and mild toxicity in trials [193,194]. Similarly, it is recommended for MCL from *L*. *panamensis* and diffuse CL from *L*. *aethiopica*, reflecting its species-specific effectiveness [195–197].

When primary treatments (pentavalent antimonials) fail, pentamidine serves as a secondary option for leishmaniasis [198]. Its efficacy, however, is inconsistent across studies: in Peru, only a 35% cure rate was reported for *L*. *braziliensis* infections, contrasting the 78% efficacy of meglumine antimoniate [199], whereas in Colombia, a study showed a 96% success rate [197]. This suggests that pentamidine’s effectiveness varies by region and *Leishmania* strain, highlighting the need for more research to define its precise therapeutic role.

Pentamidine’s clinical use is hampered by its safety profile and resistance development. Immediate reactions like hypotension, nausea, and vomiting, along with injection site pain, leukopenia, and hypoglycemia, underscore its toxicity [181]. Notably, glucose metabolism disorders affected 15.3% of patients, with a 3.6% incidence of acute kidney injury and widespread mild cardiovascular effects [200]. Resistance is also a significant issue, leading to over 30% failure rates in areas like India, necessitating dose increases that heighten toxicity risks [201]. These factors underline the urgent need for new treatments without these drawbacks to improve leishmaniasis care.

## Conclusions and outlook

Leishmaniasis, a significant NTD caused by *Leishmania* spp., challenges global health. Historically treated with pentavalent antimonials, their use has declined due to toxicity and treatment failure. Other treatments like amphotericin B, miltefosine, and paromomycin face issues of cost, safety, and efficacy across species, stifling drug development due to insufficient investment and interest. Table 1 listed a further detailed comparison of the effectiveness, limitations of use, and side effects of the discussed antileishmanial drugs. However, recent efforts aim to overcome these barriers through advances in omics, combination regimens, immunomodulatory approaches, structure-based drug design, and other novel therapies. Emphasizing multidisciplinary approaches, global collaboration, and a balance of research aims is essential for advancing antileishmanial drug development and potentially eradicating the disease. To achieve this mission, the following issues central to antileishmanial pharmacotherapy warrant thorough discussion.

**Table 1:** Summary of key features of antileishmanial drugs of interest.

Drug name	Route of administration	Effective against	Limitations and side effects	Cost of treating leishmaniasis	Insight into drug improvements
Pentavalent antimonial	Intramuscular injection or intravenous infusion [1, 214]	VL: *Leishmania donovani* (East Africa): Highly effective (94% cure rate) *Leishmania infantum* (all regions): Highly effective (97% cure rate) PKDL: ● East Africa: Good efficacy in combination with paromomycin CL: Old World species (*L*. *major*, *L*. *tropica*, *L*. *infantum*): Highly effective New World species (*L*. *braziliensis*, *L*. *panamensis*, *L*. *guyanensis*): Highly effective ML: ● New World species: Moderate efficacy, may require combination therapy	● Common side effects: Musculoskeletal pain, headache, nausea, and asthenia ● Cardiotoxicity, hepatotoxicity, nephrotoxicity, and pancreatitis as rare, and associated with cumulative doses [215–219] ● Abdominal colic, diarrhea, skin rashes, pancreatitis ● Painful to administer and prolonged treatment ● Drug resistance (in the Indian subcontinent) [1,6]	Meglumine antimoniate (Glucantime): US$ 85 per patient cured [220]	● Natural Cell-Penetrating Nanopeptide: Combined with Pentavalent Antimonial [221] ● Liposomal Encapsulation: Pentavalent Antimonials encapsulated in conventional liposomes [55–57] ● Polymer-Based Delivery Systems: Polyacryl starch microparticles containing covalently bound SSG [222] ● Cyclodextrin-Based Oral Formulation: Composition with MA and β-cyclodextrin enhances oral absorption in a murine model of CL [57] Topical Formulations for CL: Sb(V)-guanosine hydrogel highly effective against intracellular *Leishmania* amastigote [223–225] Amiodarone and Itraconazole: In hamsters, either alone or in combination, enhances glucantime activity in treating *L*. *amazonensis* lesions with no evident side effects [226]
Amphotericin B	Intravenous injection (for AmB deoxycholate and L-AmB) [80,68] or topical (for L-AmB gel) [100]	L-AmB: Showed efficacy against *Leishmania infantum* in a documented Korean case [94] Emerging resistance reported in some *L*. *martiniquensis* strains may confer cross-resistance [108] Topical L-AmB gel: Highly effective against patients with CL caused by *L*. *major* [100]	AmB deoxycholate: ● Inherent nephrotoxicity [62,68,80] ● Longer hospitalization [89] ● Infusion reactions like fever, chills [103] L-AmB: ● Milder toxicity than amphotericin B deoxycholate [62,68,80–84,88] Topical L-AmB gel: ● Mild local adverse reactions like pain, itch, erythema, and discharge [100]	US$ 659.79 (price adopted by WHO) or US$ 11,559.15 (price adopted by the Drug Regulation Board of Brazil) for treating an adult patient with VL in Brazil [227]	● Improve liposomal formulations to reduce toxicity [228] ● Short-course combination with miltefosine [111] ● Nanoparticle incorporation to improve delivery [110]
Miltefosine (trade name: Impavido)	Oral administration [116,145]	*L*. *donovani* was found to be the most sensitive, while *L*. *major* is not sensitive [116,229]	● Gastrointestinal complaints ● Teratogenicity [116,144] ● Ocular complications [144,230] ● Reversible male reproductive toxicity [116,145]	US$ 259.92 in the outpatient treatment regimen in Brazil [231]	● Combination therapy [144] ● Formulation innovation [150]
Paromomycin	Intramuscular injection; intravenous injection; topical application [165]	Strains: Efficacy against several *Leishmania* strains, including *L*. *major*, *L*. *panamensis*, *L*. *mexicana*, and *L*. *braziliensis* in varying regions [168–171] ● Advantageous regions: Africa and India [151]	● Injection-site pain [157] ● Mild side effects [157] ● Laboratory strains developed resistance [165]	Cost-effective: US$ ~10 per patient [232]	Develop some innovative formulations to enhance the efficacy and safety of paromomycin
Pentamidine	Intramuscular injection [165]	First-line recommendation for CL and MCL caused by *L*. *guyanensis*, which is endemic to Brazil, Colombia, French Guiana, and Suriname [193,194]	Efficacy varies between *Leishmania* species [165] ● Drug resistance [201]	US$ 70 for relapsed patients [233]	Developing agents devoid of resistance and toxicity issues

Given the outlined challenges, antileishmanial drug development faces critical issues, notably the suboptimal efficacy and drug resistance of current treatments, underscoring the need for safer and more effective alternatives. The adverse effects and safety concerns of drugs like pentavalent antimonials, along with the high costs and limited accessibility of treatments such as L-AmB and miltefosine, constrain their use, particularly in under-resourced areas. Furthermore, the administration routes of most existing agents, typically requiring parenteral injections, contribute to poor patient compliance and highlight the need for oral or topical options. Another key obstacle is the lack of treatment specificity against diverse *Leishmania* species and clinical manifestations, which hampers the development of tailored therapeutic regimens. To address these challenges, research advancements are crucial, particularly in the areas of combination therapies and novel drug delivery systems, such as nanoparticles, liposomes, and hydrogels, which offer promising avenues for enhancing treatment efficacy and reducing toxicity. Recent innovations in drug delivery have introduced advanced nanotechnology and liposomal carriers to boost bioavailability and mitigate toxicity. For example, pH-sensitive NLCs for AmB have been developed to enable targeted drug release under the acidic conditions typical of localized leishmaniasis lesions, thus meeting the need for localized treatments. Likewise, liposomal formulations, such as those created for AmB, enhance drug distribution and reduce side effects, proving particularly effective in the treatment of VL [62,63]. These technologies exemplify how innovations in drug delivery can expand treatment options [202] and improve outcomes for patients across various forms of the disease. Moving forward, the future of antileishmanial therapy hinges on continued innovation that addresses these diverse challenges, aiming for treatments that are not only efficacious but also safe, cost-effective, and widely accessible.

Additionally, rationale structural optimization guided by insights into drug–parasite interactions may yield derivatives with increased potency and selectivity. Developing novel formulations for diverse administration routes, including oral and topical options, could further expand therapeutic reach and access. New compounds play an important role in anti-parasitic treatment. Wyllie and colleagues have identified small molecule drugs that can inhibit the growth and reproduction of parasites by inhibiting the CRK12 enzyme [203]. As a prominent among drugs for treatment of infectious disease, macrocycles were more potent than miltefosine identified in a phenotypic screen of *Leishmania infantum* [204]. Melittin-containing fusion crystal [205], dioclea violacea lectin [206], 8-hydroxy-2-quinoline carbaldehyde derivatives [207], cyanotriazoles that rapidly cure trypanosome infections [208] also have been discovered to be used for treating leishmaniasis. Beyond traditional chemicals, newly developed gene editing methods also present opportunities to eliminate remaining parasites following therapy. To improve efficacy and reduce side effects, Lago and colleagues utilized topical rSm29 in conjunction with intravenous meglumine antimoniate for the treatment of cutaneous leishmaniasis [209]. As the conventional treatments often use drugs with high toxicity, A chitosan/collagen biomembrane, loaded with 2,3-dihydrobenzofuran can be employed for the treatment of CL [210]. Nahanji and colleagues enhanced the efficacy of fluconazole against *Leishmania major* for topical delivery using FLZ-nanoemulsions [211]. Besides, PA and AmpB together could form a promising new treatment strategy against *Leishmania* infections, offering enhanced efficacy without added toxicity [212]. Nanotechnology can enhance leishmaniasis treatment using drug-carrying nanosystems like metallic nanoparticles, liposomes, and polymeric/lipid nanoparticles, minimizing side effects, dose, and costs. Encapsulating antileishmanial drugs in nanosystems boosts bioavailability, sustained release, macrophage uptake, and target cell/tissue delivery, while enhancing efficacy and reducing toxicity [150]. Allahverdiyev and colleagues found Ag-NPs inhibited *L*. *tropica* promastigote proliferation and metabolic activity by 1.5–3× in the dark and 2–6.5× under UV light [213]. By combining strengths in parasitology, pharmacology, immunology, formulation science, and bioengineering, the next generation of antileishmanial regimens may be within reach.

The future of antileishmanial therapy looks promising, driven by innovations that broaden treatment possibilities. The development of broad-spectrum agents to combat various *Leishmania* species and manifestations is crucial. Enhancing current treatments through novel formulations and delivery systems, alongside multidisciplinary methods including immunopharmacology, gene editing, and bioengineering, could offer synergistic benefits, improving safety and efficacy. Additionally, uncovering unknown drug actions and resistance mechanisms is vital for creating targeted therapies. Achieving these advancements requires global collaboration and investment, emphasizing the need to address this neglected disease’s impact. This concerted effort could usher in a new era of improved outcomes for leishmaniasis patients.

**Key Learning Points**

**Current antileishmanial drugs and their limitations**

The review discusses the main antileishmanial drugs: pentavalent antimonials, amphotericin B, miltefosine, paromomycin, and pentamidine. Each of these drugs faces significant limitations, such as high toxicity, resistance issues, and variable efficacy across different regions and *Leishmania* species.

**Drug resistance mechanisms**

*Leishmania* parasites develop resistance through various mechanisms, including alterations in drug targets, increased efflux of drugs, and metabolic changes. Understanding these mechanisms is essential for developing new, more effective treatments.

**Advancements in drug delivery systems**

Innovative drug delivery methods, such as liposomal formulations and advanced drug delivery systems, have been developed to improve the efficacy and reduce the toxicity of antileishmanial drugs. Liposomal amphotericin B, for example, offers improved outcomes with reduced toxicity.

**Combination therapies and new approaches**

The review highlights the potential of combination therapies and new therapeutic strategies, including combination regimens, immunomodulatory approaches, and advanced drug delivery systems, to overcome the limitations of current treatments and improve patient outcomes.

**Future directions and research needs**

Continued research is needed to explore novel therapeutic targets, develop safer and more effective drugs, and implement comprehensive strategies to manage drug resistance. Collaborative efforts and multidisciplinary approaches are crucial for advancing antileishmanial pharmacotherapy and addressing the global burden of leishmaniasis.

**Five Key Papers in the Field**

1. Zulfiqar B, Avery VM. Assay development in leishmaniasis drug discovery: a comprehensive review. Expert Opin Drug Discov. 2022 Feb;17(2):151–166.
2. Altamura F, Rajesh R, Catta-Preta CMC, Moretti NS, Cestari I. The current drug discovery landscape for trypanosomiasis and leishmaniasis: Challenges and strategies to identify drug targets. Drug Dev Res. 2022 Apr;83(2):225–252.
3. Roquero I, Cantizani J, Cotillo I, Manzano MP, Kessler A, Martín JJ, McNamara CW. Novel chemical starting points for drug discovery in leishmaniasis and Chagas disease. Int J Parasitol Drugs Drug Resist. 2019 Aug;10:58–68.
4. Akhoundi M, Downing T, Votýpka J, Kuhls K, Lukeš J, Cannet A, Ravel C, Marty P, Delaunay P, Kasbari M, Granouillac B, Gradoni L, Sereno D. *Leishmania* infections: Molecular targets and diagnosis. Mol Aspects Med. 2017 Oct;57:1–29.
5. Andrade-Neto VV, Cunha-Junior EF, Faioes VDS, Pereira TM, Silva RL, Leon LL, Torres-Santos EC. Leishmaniasis treatment: update of possibilities for drug repurposing. Front Biosci (Landmark Ed). 2018 Jan 1;23(5):967–996.
